# Rocuronium Dosing by Ideal vs Total Body Weight in Obesity: A Prospective, Observational Non-inferiority Study

**DOI:** 10.5811/westjem.60713

**Published:** 2023-12-06

**Authors:** Marc McDowell, Amanda Lewandowski, Dharati Desai, Stephany Nunez Cruz, Nicole Glowacki, Alaa Sulh, Michael Cirone, Nadine Lomotan, Mary Hormese

**Affiliations:** * Department of Pharmacy, Advocate Christ Medical Center, Oak Lawn, Illinois; † Department of Pharmacy, Rush University Medical Center, Chicago, Illinois; ‡ Advocate Aurora Research Institute, Advocate Aurora Health, Wilwaukee, Wisconsin; § Department of Pharmacy, Loyola University Medical Center, Maywood, Illinois; ∥ Department of Emergency Medicine, Advocate Christ Medical Center, Oak Lawn, Illinois; ¶ University of Illinois – Chicago, Chicago, Illinois; # Department of Pharmacy, Northwest Community Hospital, Arlington Heights, Illinois

**Keywords:** rocuronium, rapid sequence intubation, intubation conditions, obesity, ideal body weight, total body weight

## Abstract

**Background:**

Providing adequate paralysis and appropriate sedation is challenging in patients with obesity during rapid sequence intubation (RSI). Pharmacokinetic parameters play an important role in dosing of rocuronium due to low lipophilicity. Rocuronium may be dosed based on ideal body weight (IBW). Current guidelines do not offer recommendations for dosing in the setting of obesity. Dosing depends on clinician preference based on total body weight (TBW) or IBW.

**Objectives:**

In this study we performed non-inferiority analysis to compare the intubation conditions, duration of paralysis, and incidence of new-onset tachycardia or hypertension after intubation in obese patients requiring RSI in the emergency department (ED).

**Methods:**

This was a single-center, prospective, observational study. Eligible for enrollment were adult patients with a TBW ≥30% IBW or body mass index ≥30 kilograms per meters squared who presented to the ED requiring RSI with the use of rocuronium. Rocuronium was dosed according to intubating physicians’ preference. Physicians completed a survey assessing intubation conditions. Height and weight used for the calculation of the dose, the dose of rocuronium, time of administration, and time of muscle function recovery were recorded. Endpoints assessed included grading of view during laryngoscopy, first-past success, and duration of paralysis.

**Results:**

In total, 96 patients were included, 54 in TBW and 42 in IBW. The TBW cohort received a mean of 1 milligram per kilogram (mg/kg) compared to 0.71 mg/kg in the IBW group. Excellent intubation conditions were observed in 68.5% in the TBW group and 73.8% in the IBW group. The non-inferiority analysis for relative risk of excellent intubation was 1.12 (*P* = 0.12, [90% CI 0.80–1.50]).

**Conclusion:**

Non-inferiority analysis suggests that IBW dosing provides similar optimal intubation conditions when compared to TBW dosing, but the noninferiority comparison did not reach statistical significance. This study was unable to show statistical non-inferiority for IBW dosing.

Population Health Research Capsule What do we already know about this issue? *About one third of adults in the US suffer from obesity. Patients with obesity pose a challenge in dosing of rocuronium in the setting of rapid sequence intubation*.

What was the research question? *This study compared rocuronium dosing based on ideal body weight (IBW) and total body weight (TBW).*

What was the major finding of the study?*Results suggest similar efficacy in optimal intubation conditions between IBW (73.8%) and TBW (68.5%, p = 0.12 [0.8–2.5]), as well as shorter duration of paralysis when dosing patients based off IBW (43 vs 71 minutes, p < 0.001).*

How does this improve population health?*Given challenges in determining a patient’s TBW, rocuronium dosing in patients with obesity may be done using their IBW, but this study failed to show the two to be statistically equivalent.*

## INTRODUCTION

### Background

Approximately one third of adults in the United States suffer from obesity.^1^ Caring for this population is becoming more common in the emergency department (ED). Patients with obesity pose a unique challenge to the successful performance of rapid sequence intubation (RSI).^2^ Factors potentially influencing RSI include neck circumference, anterior neck adipose tissue, and prevalence of concomitant lung disorder. These anatomic concerns have led to obesity being identified as an independent risk factor for difficult intubation.^3^

The ED setting poses inherent difficulty in the dosing and pharmacokinetics of RSI medications. An unfasted, unstable, or anatomically complex patient provides a true challenge in the dosing of RSI medications.^4^ Further confounding this issue are patients presenting with obesity. Weights used to dose RSI medications often rely on clinician estimations. Clinical staff in the ED have demonstrated an unreliable ability to estimate patients total body weight (TBW), with only 23% of physicians accurately estimating within 10% of actual patient weight in patients with a body mass index (BMI) >30 kilograms per meters squared.^5^ Inaccurate estimations may result in inappropriate amounts of paralytics. Underdosing may lead to difficult intubation conditions, increasing the risk of aspiration of gastric contents, airway trauma, hypoxia, and death.^6^ Conversely, supratherapeutic dosing of paralytic agents may lead to unrecognized under-sedation, resulting in patient awareness. This may increase the risk of hypertension, tachycardia, difficulty obtaining timely neurologic exam and, ultimately, post-traumatic stress disorder.^7^

Rocuronium is a routinely used neuromuscular blocking agent in the ED.^8^ It has a relatively low lipophilicity, moderate serum protein binding, and small volume of distribution compared to other commonly administered paralytics.^9^ This allows therapeutic serum levels of rocuronium to be achieved using ideal body weight (IBW), as accumulation is not expected to occur in adipose tissue. Due to the paucity of data surrounding appropriate dosing weight for rocuronium in patients with obesity, dosing remains dependent on practitioner preference. The few studies that have compared rocuronium dosing in patients with obesity typically occurred in surgical settings.^10^ ^,^ ^11^

### Goals of This Investigation

In this study our goal was to compare, via non-inferiority analysis, the intubation conditions, duration of paralysis, and incidence of new-onset tachycardia or hypertension after intubation in obese patients requiring RSI in the ED.

## METHODS

### Study Design and Setting

We conducted this prospective, observational study at a single, tertiary, community teaching ED. Study recruitment occurred from December 1, 2018–May 2, 2021. Patients were included if they presented to the ED while a clinical pharmacist was on duty, underwent RSI with rocuronium, had a BMI ≥30, or TBW ≥30% of IBW. Patients were excluded if they were under the age of 18, had known neuromuscular disease, known allergy or sensitivity to rocuronium, or concomitant medications known to interfere with neuromuscular transmission. Design of this observational study was done in alignment with the STROBE checklist. Study approval was granted by the local institutional review board.

### Interventions

Immediately prior to the procedure, the intubating physician would ask the clinical pharmacist to dose rocuronium based on IBW or TBW. The IBW dose was obtained via the pharmacist using a measuring tape bedside and the Devine formula, with dosing at 1milligram per kilogram (mg/kg). Dosing by TBW used recent chart documentation within the prior three months, measured bed weight, or estimation by the medical team. Both dosing strategies were capped at 100 mg. All patients were weighed after intubation. Upon administration of the paralytic, the pharmacist at bedside announced passage of time at 15-second intervals. Laryngoscopy was performed at the discretion of the intubating physician based on passage of time, diaphragmatic movement, and patient-specific clinical factors.

### Measurements

The pharmacist noted height and weight used for the calculation of the dose, dose of rocuronium, time of medication administration, time of intubation, need for repeat dose of paralytic, use of bougie, post-rocuronium hypoxia defined as a pulse oximeter reading of <90%, and the education level of the intubating physician. Time of muscle function recovery was observed and documented by either the nurse or clinical pharmacist present at the bedside. This was determined by spontaneous movement or spontaneous breathing. Post-intubation sedation selection and dosing was initiated at the discretion of the intubating physician. Data collection was performed prospectively, and abstractors were not blinded to the study hypothesis.

### Outcomes

**Figure 1.**
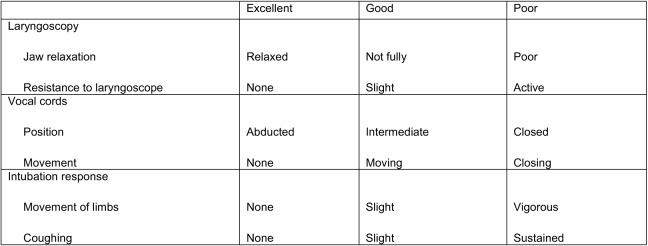
Good Clinical Research Practice Guidelines Airway Assessment. Intubating conditions: excellent = all qualities excellent; good = all qualities are excellent or good; poor = any quality is poor.

The primary outcome measured was the incidence of poor, good, or excellent intubation conditions, measured using a validated nine-point Good Clinical Research Practice Guidelines Airway Assessment survey (Figure 1), which was provided to the intubating physician.^12^ Secondary endpoints included first-pass success, duration of paralysis, and incidence of suboptimal sedation defined as post-intubation systolic blood pressure and/or tachycardia greater than 30% of baseline.

### Statistical Analysis

Using non-inferiority analysis, we analyzed the null hypothesis that intubating conditions (excellent/good/poor) in patients with obesity undergoing RSI in the ED did not differ based on use of IBW or TBW to calculate rocuronium doses. This resulted in an estimated subject sample size of 90 patients, on the assumption of “excellent” conditions occurring in 80% of intubations, with a 10% non-inferiority margin, and 80% power. We used the Farrington-Manning method for the non-inferiority analysis. Descriptive statistics were calculated for all variables and presented as mean ±SD for continuous variables and count/percentages for categorical variables. We made comparisons between groups for all outcomes using chi-square or Fisher exact tests as necessary for categorical data and Student *t*-tests or Mann-Whitney tests as appropriate for all continuous data. All tests were two-tailed and a *P*-value of 0.05 was considered statistically significant in all analyses.

## RESULTS

### Characteristics of Study Subjects

**Figure 2.**
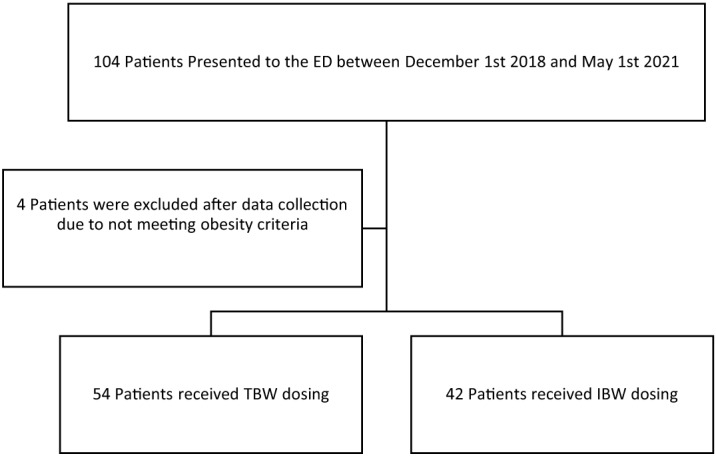
CONSORT (Consolidated standards of reporting trials) diagram. *ED*, emergency department; *TBW*, total body weight; I*BW*, ideal body weight.

We collected data on 104 patients. Eight were excluded as they did not meet pre-specified obesity criteria (Figure 2). The remaining 96 patients were included for analysis. The TBW cohort included 54 subjects, while 42 were included in the IBW arm. (Summary of demographics can be found on in Table 1). Median actual body weight was similar in the TBW group (98.0 kg) as compared to the IBW group (98.9 kg). The TBW arm consisted of 51.9% females as compared to 64% in the IBW arm. Median age was similar between both cohorts, 60 and 65 years in TBW and IBW, respectively. Etomidate was the most routinely selected induction agent (92.6% and 90.5%). Median rocuronium dose used was 100 mg compared to 70 mg, respectively (*P* < 0.001) (Table 1). Median dosing weight used was 98 kg in TBW and 70 kg in the TBW (*P* < 0.001). Mean rocuronium dose based on actual body was 1 mg/kg (±0.01) in TBW and 0.71 mg/kg (±0.12) in the IBW cohort.

**Table 1.** Patient baseline demographics.

Baseline characteristics	TBW (n = 54)	IBW (n = 42)	*P*-value
Age, median (IQR)	60 [51–76]	65 [57–81]	0.70
Height (in), median (IQR)	67 [64–71]	68 [66–71]	0.07
Weight (kg), median (IQR)	98 [82–106]	98.9 [83–120]	0.74
Dosing weight (kg), median (IQR)	98 [82–100]	70 [60.0–75.0]	<0.001
Female gender, No. (%)	28 (51.9)	27 (64.3)	0.11
Sedative agent			
Etomidate	50 [92.6%]	38 [90.5)	0.93
Ketamine	3 [5.6%]	3 [7.1)	
None	1 [1.9%]	1 [2.4)	
Intubating physician, n (%)			
PGY1	12 (22.2)	7 [16.7)	0.92
PGY2	13 (24.1)	10 [23.8)	
PGY3	23 (42.6)	20 [47.6)	
Attending	6 (11.1)	5 [11.9)	
Use of bougie	5 (11.1)		
Rocuronium dose (mg), median (IQR)	100 [90–100]	70 [60–80]	<0.001

*TBW*, total body weight; *IBW*, ideal body weight; *IQR*, interquartile range; *kg*, kilogram; *PGY*, postgraduate year; *mg*, milligram.

### Main Results

The primary outcome, excellent intubation condition, was observed in 68.5% of patients in the TBW group and 73.8% in the IBW group (Table 2). Non-inferiority analysis for relative risk of excellent intubation was 1.12 (*P* = 0.12, 90% confidence interval [CI] 0.80–1.50) (Table 3). Pertinent secondary outcomes analyzed included first-pass success which was achieved in 92.6% of the TBW cohort compared to 85.7% in IBW cohort (*P* = 0.27). Duration of paralysis, measured by median time to muscle recovery, was observed at 71 minutes and 43 minutes, respectively (*P* < 0.001). Hypoxia was observed in 17.8% of subjects in TBW arm compared to 3.45% in the IBW cohort (*P* = 0.07). Incidence of post-intubation hypertension was 44.4% and 23.8% (*P* = 0.04). The incidence of new-onset tachycardia was similar between groups, 35.2% and 33.3%. Level of physician training did not differ between groups (*P* = 0.92). An ad hoc analysis was performed comparing the combination of excellent and good views compared to a poor view. All patients in the IBW were assessed as having a good or excellent view compared to 94.4% in the TBW arm (*P* = 0.04, 90% CI 0.94–2.16).

**Table 2.** Intubation condition.

Outcomes	TBW (n = 54)	IBW (n = 42)	*P*-value
Good Clinical Research Practice Guidelines Airway Assessment, n (%)			
Excellent	37 (68.5)	31 (73.8)	0.3
Good	14 (25.9)	11 (26.2)	
Poor	3 (5.6)	0 (0)	
First-pass success, n (%)	50 (92.6)	36 (85.7)	0.27
Post-rocuronium hypoxia, n (%)	8 (17.8)	1 (3.45)	0.07
Duration of paralysis (min), median (IQR)	71 (57–96)	43 (39–54)	<0.001
Incidence of post-intubation hypertension, n (%)	24 (44.4)	10 (23.8)	0.04
Incidence of post-intubation tachycardia, n (%)	19 (35.2)	15 (33.3)	0.85

*TBW*, total body weight; *IBW*, ideal body weight; *IQR*, interquartile range.

**Table 3.** Non-inferiority analysis.

Outcomes	TBW (n = 54)	IBW (n = 42)	*P*-value	95% CI
Good Clinical Research Practice Guidelines Airway Assessment, n (%)				1.12 [0.80–1.50]
Excellent	37 (68.5)	31 (73.8)	0.12	
Good/poor	17 (31.5)	11 (26.2)		

*TBW*, total body weight; *IBW*, ideal body weight; *CI*, confidence interval.

## DISCUSSION

The results of the study suggest that there is no difference in good/excellent intubation conditions when rocuronium is dosed based on IBW or TBW. Time to muscle recovery was statistically significant between IBW and TBW dosing. There was a direct correlation to longer duration of action of rocuronium in patients who were dosed based on their TBW. Post-intubation hypertension occurred more frequently in the TBW cohort, which may indicate underdosing of sedation. Curiously, hypoxia was found to be higher in the TBW cohort with an incidence approaching 18%. While not statistically significant this finding is greater than anticipated without obvious evidentiary explanation.

In supporting studies, Pappal et al assessed the prevalence of awareness with paralysis in ED patients who received mechanical ventilation.^6^ They found awareness with paralysis was significantly higher in patients who were exposed to rocuronium. Levin et al assessed the association of rocuronium dosing on first-attempt success and adverse outcomes among ED patients.^8^ Their results demonstrated a higher incidence of first-pass success in rocuronium doses ≥1.4 mg/kg. This is in contrast to our findings. Considerations for this discrepancy may be due to the conventional “capping” of doses at our institution of 100 mg and the relative body habitus from the Levin et al cohort. In their ≥1.4 mg/kg arm, the documented weight was remarkably low with a mean of 67.3 kg, and only 13.2% of the 2,302 subjects met obesity criteria. Meyhoff et al conducted a study in which rocuronium dosing was compared in morbidly obese patients scheduled for laparoscopic banding or gastric bypass. Dosing was based on either ideal body weight, 20% of corrected body weight (CBW) and 40 % of CBW.^10^ Similar to our results, they report IBW provided a shorter duration of action without significantly prolonging onset time or compromising intubation conditions. However, this cohort drastically differed from ours as it occurred in a controlled surgical setting and did not include critically ill patients.

## LIMITATIONS

The main limitations of this study include the observational nature of the study design, the use of a convenience sample, the fact that chart abstractors were not blinded to the study, and a lack of standardized time from rocuronium administration to laryngoscopy. First-pass success is a complicated outcome fraught with many contributing factors, not necessarily related to paralytic dose. Variability exists in the scoring of ideal conditions, as it may depend on comfort level with the procedure itself. Lastly, the patients’ duration of paralysis was based on a subjective nature of observation by clinicians.

## CONCLUSION

The results of this study suggest that, while similar intubation conditions appear to be produced with ideal body weight or total body weight dosing, this study did not show statistically significant non-inferiority between TBW and IBW. Additionally, a shorter duration of paralysis was observed when dosing was based on ideal body weight. However, additional prospective studies of an interventional nature are needed to determine optimal dosing of rocuronium in obesity.
